# The Switching of the Type of a ROS Signal from Mitochondria: The Role of Respiratory Substrates and Permeability Transition

**DOI:** 10.3390/antiox13111317

**Published:** 2024-10-29

**Authors:** Alexey G. Kruglov, Anna B. Nikiforova

**Affiliations:** Institute of Theoretical and Experimental Biophysics, Russian Academy of Sciences, Institutskaya 3, Pushchino 142290, Moscow Region, Russia; office@iteb.ru

**Keywords:** mitochondria, superoxide anion, hydrogen peroxide, kinetics, redox signaling, OXPHOS substrates

## Abstract

Flashes of superoxide anion (O_2_^−^) in mitochondria are generated spontaneously or during the opening of the permeability transition pore (mPTP) and a sudden change in the metabolic state of a cell. Under certain conditions, O_2_^−^ can leave the mitochondrial matrix and perform signaling functions beyond mitochondria. In this work, we studied the kinetics of the release of O_2_^−^ and H_2_O_2_ from isolated mitochondria upon mPTP opening and the modulation of the metabolic state of mitochondria by the substrates of respiration and oxidative phosphorylation. It was found that mPTP opening leads to suppression of H_2_O_2_ emission and activation of the O_2_^−^ burst. When the induction of mPTP was blocked by its antagonists (cyclosporine A, ruthenium red, EGTA), the level of substrates of respiration and oxidative phosphorylation and the selective inhibitors of complexes I and V determined the type of reactive oxygen species (ROS) emitted by mitochondria. It was concluded that upon complete and partial reduction and complete oxidation of redox centers of the respiratory chain, mitochondria emit H_2_O_2_, O_2_^−^, and nothing, respectively. The results indicate that the mPTP- and substrate-dependent switching of the type of ROS leaving mitochondria may be the basis for O_2_^−^- and H_2_O_2_-selective redox signaling in a cell.

## 1. Introduction

In recent years, the area of research associated with redox signaling in cells in normal and pathological states has been actively developing [1,2]. The data obtained indicate the complexity and ramification of the system of redox signaling, which includes subsystems of production and transformation of ROS, amplification/weakening of a redox signal, redox sensors, and redox-dependent effectors that provide a local or generalized cellular response [3,4]. Presently, more than 200 mammalian proteins are known whose activity can be regulated by the redox state of thiol groups or the level of ROS. It is proposed to call this community the “redoxome” [5].

Mitochondria are one of the main sources of ROS in the majority of cell types except specialized ROS producers: leukocytes and macrophages [6]. ROS produced by mitochondria contribute to the development of many pathologic states including cancer, cardiovascular diseases, diabetes, neurological disorders, muscular dystrophy, etc. [7]. One of the most interesting phenomena associated with ROS generated by mitochondria is the so-called superoxide anion (O_2_^−^) flashes discovered about 15 years ago [8,9,10,11,12]. An O_2_^−^ flash is a sharp short-term acceleration of O_2_^−^ production by individual mitochondria in the cell [10,12]. As a rule, the time to achieve the maximum O_2_^−^ production in a single mitochondrion does not exceed a few seconds, while the decay time is about 20 s [10,11,12,13,14]. O_2_^−^ flashes can occur spontaneously, but their frequency can increase, and their generation can be synchronized under certain conditions. In particular, oxidants, Ca^2+^ and other inducers of mPTP, and respiratory substrates increase the frequency of flashes, while antioxidants, uncouplers and inhibitors of the electron transport chain (ETC), and mPTP blockers reduce the frequency of flashes or completely abolish them [10,11,12,13,14]. Moreover, flashes can propagate from mitochondria to mitochondria, causing the permanent or temporary depolarization, i.e., a “wave of dysfunction” [11,15,16]. Though flashes are often associated with transient or long-term mPTP opening, this condition is not obligatory [17,18]. It was demonstrated that flashes can appear in intact mitochondria [11,14] and, presumably, play an important role in cell physiology and pathophysiology. Flashes participate in cell excitation [19,20] and regulation of cell differentiation, proliferation [21,22,23,24], muscular development [14], and animal longevity [25], as well as in the progress of muscular diseases [26], ischemia-reperfusion-dependent injury [9], amyotrophic lateral sclerosis [27], and oxidant-induced apoptosis [28]. However, in recent years, the flow of works devoted to the study of O_2_^−^ flashes has been gradually drying up. It can be assumed that this is due to a serious criticism of the methods used to detect O_2_^−^ flashes, on the one hand, and the paradoxical mechanism of their generation, on the other.

The data on O_2_^−^ flashes in mitochondria were obtained predominantly using the O_2_^−^-sensing matrix-targeted circularly permuted yellow fluorescent protein (mt-cpYFP) [8,29] and the cationic derivative of hydroethidine Mito-SOX red [11,13,30]. Another matrix-targeted protein, the circularly permuted green fluorescent protein (pericam), was applied for the recording of O_2_^−^ flashes in mouse skeletal muscle mitochondria [11]. In addition, uncharged and relatively hydrophilic 2,7-dichlorodihydrofluorescein diacetate was used for detecting H_2_O_2_ flashes in the cell cytosole [12].

However, the ability of the mt-cpYFP protein to detect O_2_^−^ was questioned in several studies. In particular, it was shown that an increase in the fluorescence of the detector can reflect the alkalization of the matrix, but not the generation of O_2_^−^ [31,32,33]. In addition, another O_2_^−^ probe, Mito-SOX red, being sensitive to the mitochondrial membrane potential (ΔΨm), relatively non-specific, and reactive toward DNA, also cannot be a reliable detector in mitochondria when their functional state changes and, especially, when the mPTP opens [34,35].

The proposed mechanism for the emergence of O_2_^−^ flashes also raises a number of questions. First, it is widely believed that “mitoflashes are quantal bursts of ROS production accompanied by the modest matrix alkalinization and depolarization of the mitochondrial membrane potential” [34]. However, mitochondrial depolarization, by definition, should cause the acidification of the matrix, especially if the mPTP is irreversibly opened, since, in this case, the protons pumped out by the pumps of the respiratory chain immediately return to the matrix. Theoretically, ΔΨm dissipation and the alkalization of the matrix may co-exist during the transient mPTP opening or stochastic short-term drops in ΔΨm [36] in accordance with the mechanism proposed by Schwarzlander et al.: stochastic depolarization causes the activation of proton pumps, which, in turn, induces matrix alkalization and a burst of ROS [37]. However, it was shown in a model of UCP-3 knockout that the burst of O_2_^−^ and alkalization can be separated [29]. Moreover, this mechanism cannot explain the generation of O_2_^−^ flashes upon permanent mPTP opening, since a large pore will prevent the formation of the pH gradient across the inner mitochondrial membrane (IMM). Second, flashes require the presence of respiratory substrates, i.e., redox centers must be reduced. However, both inhibitors, which cause the complete reduction of certain segments of the ETC, and uncouplers, which induce the full oxidation of electron carriers, inhibit O_2_^−^ flashes [8]. The question arises: How is the degree of reduction of redox centers related to the intensity of O_2_^−^ production? Third, experiments with isolated mitochondria demonstrated that the rates of ROS production are maximum upon good mitochondrial coupling and at high ΔΨm [38,39,40]. Thus, the mechanism of O_2_^−^ flashes upon mitochondrial depolarization is unclear.

Trying to resolve the paradox of the mechanism of O_2_^−^ flashes, we have previously shown that the O_2_^−^ burst can be induced in mitochondrial suspension upon permeabilization of the IMM due to the opening of mPTP or the incorporation of a pore-forming peptide [41,42]. Using uncharged and relatively hydrophilic 3,7-dihydro-2-methyl-6-(4-methoxyphenyl)imidazol[1,2-a]pyrazine-3-one (MCLA), whose accumulation in intact mitochondria is limited but which can pass through the large pores in the IMM, we demonstrated that added NADH and, especially, NADPH strongly stimulated O_2_^−^ bursts, which occur only after considerable oxidation of pyridine nucleotides [42]. However, we were unable to reliably compare the kinetics of O_2_^−^ and H_2_O_2_ generation since pyridine nucleotides (which were added to permeabilized mitochondria at high concentrations) can interfere with the oxidation of fluorogenic substrates by horseradish peroxidase (HRP) [43].

In the present work, we compared the kinetics of the release of O_2_^−^ and H_2_O_2_ from isolated mitochondria in the presence of endogenous and exogenous substrates of different mitochondrial dehydrogenases. We assessed the effect of the concentration of added substrates and mPTP opening on these processes. The data obtained indicate that the opening of the mPTP and the presence of substrates cause a bidirectional switch of the type of the ROS signal (O_2_^−^ or H_2_O_2_) emitted by mitochondria to the medium. The possible mechanism of this phenomenon and its probable physiological significance are discussed.

## 2. Materials and Methods

### 2.1. Materials

ADP (sodium salt) (A2754), ATP (disodium salt hydrate) (A7699), bovine serum albumin (BSA) (A7030), FCCP (C2920), CATR (C4992), DMSO (276855), DNP (D198501), 4-(2-hydroxyethyl)piperazine-1-ethanesulfonic acid (HEPES) (H3375), sucrose (S7903), succinate (S3674), Trizma Base (93352), Ampliflu™ Red (AR), ethylene glycol-bis(2-aminoethylether)-*N*,*N*,*N*′,*N*′-tetraacetic acid (EGTA), glutamate, 2-oxoglutarate, malate, 3-hydroxybutyrate, mannitol, MCLA, myxothiazol, NADH, NAD, potassium peroxide, pyruvate, rotenone (R8875), and superoxide dismutase (SOD) were obtained from the Sigma-Aldrich Corporation (St. Louis, MO, USA). Other chemicals were of analytical grade and were purchased from local suppliers.

### 2.2. Preparation of Mitochondria

All manipulations with animals before the isolation of the liver were performed in accordance with the Helsinki Declaration of 1975 (revised in 1983), the national requirements for the care and use of laboratory animals, and protocol 26/2024 of 18 March 2024 approved by the Commission on Biological Safety and Bioethics of the Institute of Theoretical and Experimental Biophysics, Russian Academy of Sciences (ITEB RAS). Rat liver mitochondria (RLM) were isolated by a standard procedure [44] with minor modifications [45]. Adult male Wistar rats (200–250 g) were decapitated after anesthesia with CO_2_. The liver was homogenized in ice-cold isolation buffer containing 220 mM mannitol, 70 mM sucrose, 1 mM EGTA, 0.3% BSA, and 10 mM HEPES-Tris (pH 7.4). The homogenate was centrifuged at 600× *g* for 10 min at 4 °C, and the supernatant fraction was then centrifuged at 7000× *g* for 15 min to sediment mitochondria. The RLM were washed three times (7000× *g* for 20 min) in the above medium without EGTA and BSA (1× washing medium). The final mitochondrial pellet was suspended in the washing medium to yield 70–80 mg protein/mL and kept on ice until used. The total mitochondrial protein was determined by the Biuret method using BSA as a standard [46]. All measurements unless otherwise stated were performed at 37 °C using the standard KCl-based medium (KCl-BM): 125 mM KCl, 20 mM mannitol, 10 mM HEPES (pH 7.3), 2 mM KH_2_PO_4_, and 2 mM MgCl_2_.

### 2.3. Measurement of Oxygen Uptake

Isolated RLM (1 mg protein/mL) were incubated at 25 °C in the standard KCl-BM supplemented with substrates of complex I (5 mM glutamate and 5 mM malate) (GM) or complex II (5 mM succinate) in the presence of 2 μM rotenone (SR). Oxygen uptake was measured with a Clark-type electrode using an Oroboros Oxygraph-2 k respirometer (Innsbruck, Austria). In order to assess V_3_ and V_4_ respiration rates, 500 μM ADP was added to resting RLM (V_2_). The respiratory control coefficient (V_3_/V_4_) for RLM taken for experiments was ≥6 for GM and ≥5 for SR.

### 2.4. Recording of Mitochondrial Swelling

The opening of mPTP in isolated RLM was assessed from the initiation of EGTA- and CsA-sensitive high-amplitude swelling. Mitochondrial swelling was determined by measuring a decrease in absorbance at 550 (A_550_) or 535 nm (A_535_) in suspension using the plate readers Infinite 200 Tecan and Infinite 200 Tecan Pro (Groedig, Austria), respectively, and 96-well plates. Other details are given in the figures and figure legends.

### 2.5. Assessment of ROS Production

RLM were placed in standard KCl-BM without substrates, and the suspension was immediately poured into two tubes with either MCLA at indicated concentrations or with 40 µM AR and HRP (3 U/mL). The suspensions were then distributed into the wells of plates for luminescence and fluorescence measurements, which contained substrates and other additions, as specified in figure legends, and analyzed in parallel using two plate readers.

### 2.6. H_2_O_2_ Release

Resorufin accumulation (which linearly depends on H_2_O_2_ production) was traced at excitation and emission wavelengths of 530 and 595 nm. For the quantitative measurement of H_2_O_2_, fluorescence was calibrated by the addition of an excess of H_2_O_2_ (100 μM final concentration) at the beginning and at the end of the recording to several wells. This was done to assess the rate of the conversion of AR/resorufin to non-fluorescent product [47] and thus to deduce the fluorescence of 40 µM resorufin at zero time. The rate of H_2_O_2_ release from RLM was calculated for each point of the record except the first as an increment in resorufin concentration per minute per mg of mitochondrial protein: R = Increment in fluorescence (ΔAU) · 40 · Fluorescence of 40 μM resorufin^−1^ (AU)·nmol·mL^−1^·mg protein^−1^.

### 2.7. O_2_^−^ Release

Since O_2_^−^ flashes usually occur during a spontaneous drop in ΔΨm, we used MCLA for O_2_^−^ detection. MCLA is a highly sensitive O_2_^−^ probe that requires single-electron transfer to some oxidant for activation [48]. The resulting radical reacts with O_2_^−^ with a high rate and selectivity. The rate constant is about 2.54 × 10^8^ M^−1^ s^−1^ [49], which is only an order of magnitude lower than that of SOD [50]. The relative chemiluminescence intensity of MCLA in reaction with O_2_^−^, H_2_O_2_, ^1^O_2_, and NO was determined to be 230,000, 0.4, 1400, and 17,000, respectively [51]. The product of the reaction is an unstable anionic dioxetanone, which is immediately decarboxylated to form oxy-MCLA in an excited state, emitting a quantum of blue chemiluminescence upon transition to the ground state. A quantum yield of the MCLA chemiluminescence in aqueous solutions is in the range from 0.0079 to 0.066 [52]. Both O_2_^−^-sensing MCLA radical and oxy-MCLA are uncharged [48,53], which makes MCLA-derived chemiluminescence (MDCL) independent of ΔΨm.

In the present study, MDCL was recorded approximately once a minute. Each value on the curve is the mean ± S.D. of three integrations of luminescence for 900 ms expressed in arbitrary units. In order to separate relatively bright spontaneous O_2_^−^-insensitive MDCL [53] from O_2_^−^-sensitive MDCL, some wells contained SOD at indicated concentrations. Since MDCL in solution is effectively quenched by sulfur-containing compounds [54], we excluded sulfur-containing antioxidants from the experimental protocol. It should be stressed that MDCL reflects the quantity of photons emitted by the excited oxy-MCLA within the 900 ms-period of luminescence accumulation at each experimental point (AU accumulated per 900 ms). Thus, MDCL is the measure of the rate of O_2_^−^ production. As the rate of O_2_^−^ production by mitochondria and, consequently, the intensity of MDCL were not constant during long incubation, in order to assess the net O_2_^−^ production for a long period, we integrated MDCL values within initial 60 min interval of incubation (Σ(MDCL)_0–60 min_, AU).

### 2.8. Statistical Analysis

Representative data from 3 to 20 independent experiments are given. The values on all swelling/shrinkage and ΔΨm curves are the means ± SEM for three wells (*n* = 3). Statistical significance (*p*) was determined using Student’s *t*-test.

## 3. Results

### 3.1. Effect of Respiratory Substrates and Permeability Transition Pore Inhibitors on the Release of O_2_^−^ and H_2_O_2_ from Mitochondria

**Figure 1:**
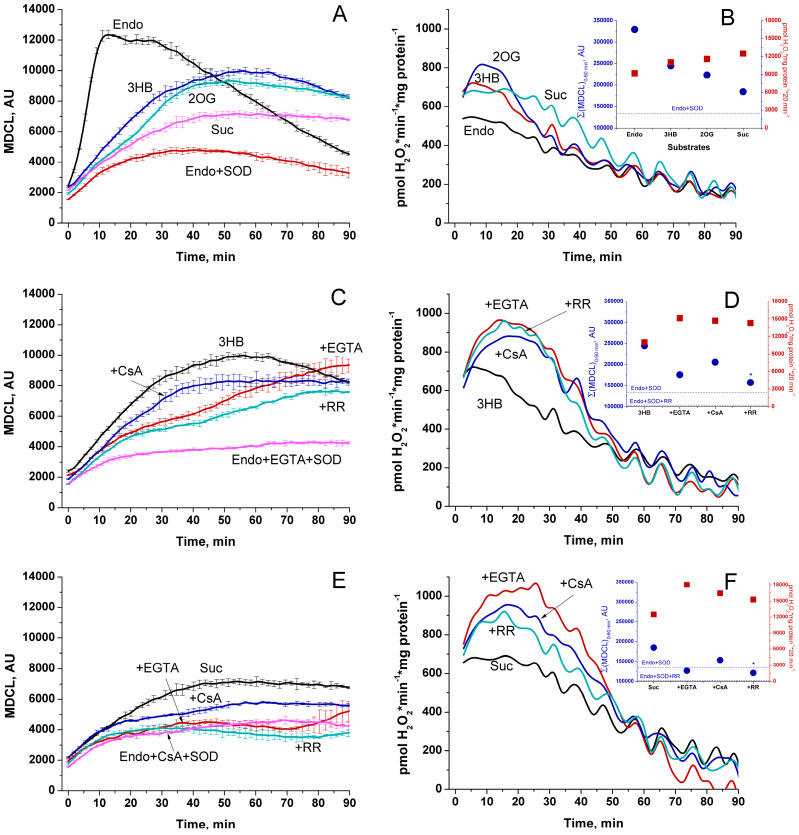
Effect of respiratory substrates and mPTP inhibitors on the release of O_2_^−^ (**A**,**C**,**E**) and H_2_O_2_ (**B**,**D**,**F**) from RLM. RLM (0.75 mg protein/mL) were placed in the standard KCl-BM without substrates and EGTA (the concentration of free Ca^2+^ was ~15 µM, as indicated by titration with EGTA), and the suspension was immediately poured into two tubes with either 15 µM MCLA or 40 µM AR and HRP (3 U/mL). The suspensions were then distributed into the wells of plates for luminescence and fluorescence measurements and analyzed in parallel using two plate readers. Where indicated, the wells contained 5 mM 3-HB, 5 mM 2-OG, 5 mM Suc, 1 mM EGTA, 2 µM RR, 1 µM CsA, and SOD (100 U/mL). Panels (**A**,**C**,**E**) show the MDCL accumulated over 900 ms at each point on the curve and expressed in AU. Points on the curves are the means ± standard deviation (*n* = 3) of three technical replicates. Panels (**B**,**D**,**F**) show the rate of H_2_O_2_ production (pmol·min^−1^·mg protein^−1^). Points on the curves are the means of three technical replicates (*n* = 3). Inserts in the panels are the data of cumulative MDCL (Σ(MDCL)_0–60 min_, AU) and H_2_O_2_ production per hour. The figure shows one representative experiment of at least five similar ones.

Initially, we studied the effect of respiratory substrates at saturating concentrations on the kinetics of the release of O_2_^−^ and H_2_O_2_ from RLM (Figure 1A and Figure 1B, respectively). The release of O_2_^−^ was assessed as SOD-sensitive MDCL. The rate of H_2_O_2_ release was determined by measuring the increment in resorufin fluorescence per mg protein per minute. A parallel study of O_2_^−^ and H_2_O_2_ release revealed several interesting features. First, the highest rate of O_2_^−^ release occurred when mitochondria oxidized endogenous substrates (Endo) (Figure 1A). In contrast, the rate of H_2_O_2_ release was the least and decreased with time (Figure 1B). Second, the highest rate of H_2_O_2_ release was recorded in the first minutes of incubation (presumably, when mitochondrial coupling was at its maximum (Supplementary Figure S1)), while the maximum in MDCL was postponed. Third, respiratory substrates, namely 3-hydroxybutyrate (3-HB), 2-oxoglutarate (2-OG), and succinate (Suc) (all at a concentration of 5 mM) (Figure 1A–F), as well as GM, pyruvate (Pyr), and SR (Supplementary Figure S2) suppressed the release of O_2_^−^ and stimulated the release of H_2_O_2_. Since O_2_^−^ penetrates through lipid membranes more poorly than H_2_O_2_, the strong O_2_^−^ release from RLM, which oxidizes endogenous substrates, may be connected with a faster mPTP opening (either transient or permanent).

The data presented in Figure 1C–F suggest that this assumption may be correct. Indeed, the antagonists of mPTP opening, namely the Ca^2+^ chelator EGTA and the inhibitors of cyclophilin D isomerase cyclosporine A (CsA) and of the mitochondrial Ca^2+^ uniporter ruthenium red (RR), suppressed the O_2_^−^ release and delayed its maximum (Figure 1C,E). In contrast, the inhibitors increased the rate of H_2_O_2_ release and made it last longer. These data can be explained by the improved mitochondrial coupling and a higher degree of reduction of redox centers of the ETC (Supplementary Figure S1), as well as by a worse permeability of the IMM to O_2_^−^ upon the inhibition of mPTP opening. In all of the above cases, the rates of the release of O_2_^−^ and H_2_O_2_ mirrored each other (corrected for the effect of RR on MDCL due to the intense staining of the inhibitor), indicating that the redox signal leaving the mitochondria switches from O_2_^−^ to H_2_O_2_ and vice versa.

### 3.2. Composition of an MDCL Signal in a Mitochondrial Suspension

**Figure 2:**
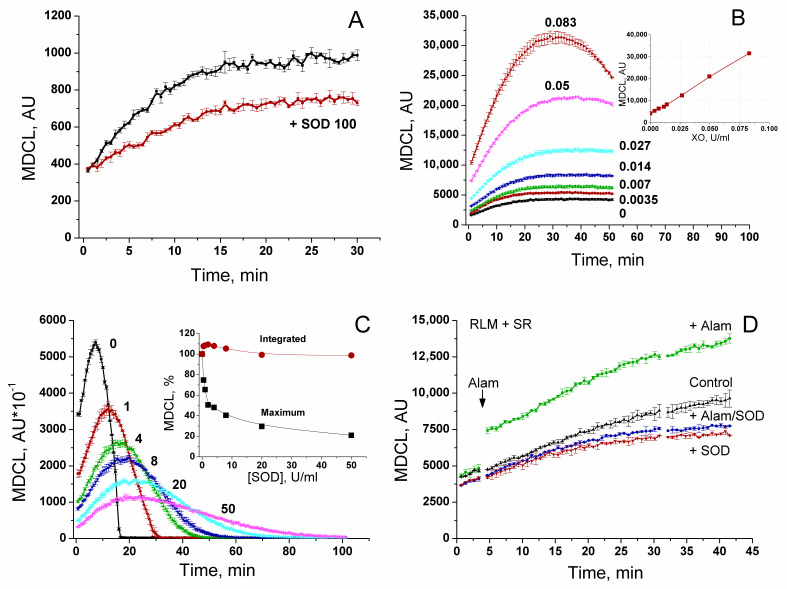
Modulation of an MDCL signal by O_2_^−^ and SOD in solution and mitochondrial suspension. (**A**) Standard KCl-BM contained 10 μM MCLA and, where indicated, SOD 100 U/mL. (**B**) The medium contained 20 μM MCLA, 400 μM xanthine, and 0–0.083 U/mL of XO. The insert shows the dependence of maximum MDCL on the concentration of XO. (**C**) Medium contained 15 μM MCLA, 400 μM xanthine, 0.15 U/mL of XO, and SOD at an indicated concentration (0–50 U/mL). The insert shows the dependence of the maximum and the integrated (for 105 min) MDCL on the concentration of SOD. (**D**) The medium contained 5 mM Suc, 1 mM EGTA, 15 μM MCLA, rotenone (2 μg/mL), and, where shown, SOD (100 U/mL). RLM (0.5 mg/mL) were added just before measurements. The arrow shows the addition of alamethicin (40 μg/mg protein). Points on the curves are the means ± standard deviation (*n* = 3) of three technical replicates. Panels show the representative data of at least three similar experiments.

In order to reveal the relationship between the level of MDCL and the rate of O_2_^−^ generation, we studied the effect of the O_2_^−^ scavenger SOD and the O_2_^−^ generator xanthine oxidase (XO) on MDCL in solution (Figure 2). Figure 2A shows that spontaneous O_2_^−^ generation in solution may be responsible for the minor, SOD-sensitive, portion of MDCL. The rest of MDCL is SOD-insensitive and can be referred to as basal (MDCL_b_). Nevertheless, XO at increasing concentrations caused a linear enhancement in MDCL (Figure 2B), indicating the linear dependence of MDCL on the O_2_^−^ concentration. In solutions with XO, the maximum MDCL was highly sensitive to added SOD (Figure 2C). However, the integrated MDCL (Figure 2C, insert) was affected to a minor extent, demonstrating that SOD effectively competes with MCLA for O_2_^−^ and thus protects the probe from oxidation. In a mitochondrial suspension, the recorded total MDCL might comprise several constituents: MDCL_b_ (SOD-insensitive and O_2_^−^-independent), SOD-sensitive O_2_^−^-dependent MDCL in solution between mitochondria (MDCL_Ext_), SOD-insensitive O_2_^−^-dependent MDCL in the intermembrane space (MDCL_IMS_), and, probably, SOD-insensitive O_2_^−^-dependent MDCL in the matrix (MDCL_Mtx_). (In all cases, we are talking about the added SOD.) Figure 2D shows that the microbial peptide alamethicin, capable of forming large pores (permeable to ~2.2 kDa substances) in mitochondrial membranes [41] and ensuring free passage of MCLA and O_2_^−^ through the IMM, caused a sharp increase in MDCL in the mitochondrial suspension. The addition of SOD-suppressed MDCL in both intact and permeabilized mitochondria to the same level. Thus, the contribution of MDCL_Mtx_ to total MDCL in the suspension of intact RLM seems negligible, presumably due to a slow penetration of MCLA through the intact IMM.

### 3.3. mPTP Opening Switches the Type of a ROS Signal Emitted by Mitochondria

**Figure 3:**
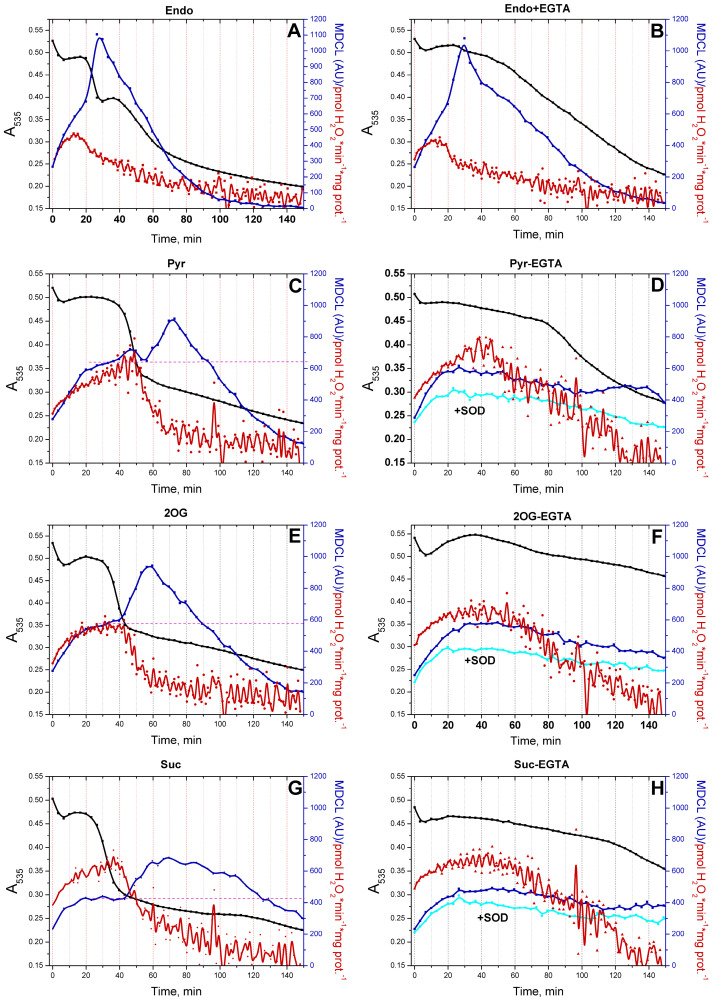
mPTP opening switches the O_2_^−^/H_2_O_2_ redox signal coming from mitochondria. RLM (0.75 mg protein/mL) were placed in the standard KCl-BM without added respiratory substrates but supplemented with 10 μM EGTA (the concentration of free Ca^2+^ was ~5 µM, as indicated by titration with EGTA). The suspension was immediately poured into two tubes with either 15 µM MCLA or 40 µM AR plus HRP (3 U/mL), transferred to the wells of plates for absorbance and fluorescence measurements, and analyzed as in Figure 1. Where indicated, the wells contained no added substrates (**A**,**B**), 5 mM Pyr (**C**,**D**), 5 mM 2-OG (**E**,**F**), 5 mM Suc (**G**,**H**), 1 mM EGTA (**B**,**D**,**F**,**H**), and SOD (100 U/mL) (**D**,**F**,**H**). In all panels, black, red, and blue lines indicate changes in absorbance, H_2_O_2_ production, and MDCL, respectively. In panels (**D**,**F**,**H**), cyan lines are MDCL in the presence of SOD. Pink dot lines show the approximate plateau of MDCL before mPTP opening. Points on the curves are the means (*n* = 3) of three technical replicates. The figure shows one representative experiment of at least five similar ones.

In order to demonstrate unequivocally that mPTP opening can switch the type of a ROS signal from mitochondria, we conducted a series of experiments in which MDCL and mitochondrial swelling (a consequence of mPTP opening, assessed as a decrease in A_535_ or A_550_) were recorded in the same wells, and H_2_O_2_ production was measured in parallel (Figure 3). In this case, the points on the luminescence curves lagged behind the points on the absorption curves by approximately 1 min. In the presence of the added respiratory substrates Pyr, 2-OG, and Suc, (all at a concentration of 5 mM) (Figure 3C, Figure 3E and Figure 3G, respectively) and in RLM-oxidizing Endo substrates in the absence of 1 mM EGTA (Figure 3A), O_2_^−^ bursts followed the mPTP opening. Again, the intensity of the bursts was a mirror image of the intensity of H_2_O_2_ release. The inhibition of mPTP opening and mitochondrial swelling at a high EGTA concentration strongly suppressed O_2_^−^ bursts in the presence of all respiratory substrates but Endo. In the latter case, EGTA suppressed the swelling, but O_2_^−^ bursts occurred approximately at the same time as in its absence. In the presence of all substrates but Endo, the inhibition of mPTP opening increased the time of H_2_O_2_ production. Thus, both mPTP opening and, probably, respiratory substrates regulate the O_2_^−^ release from mitochondria.

### 3.4. mPTP Antagonists Do Not Prevent O_2_^−^ Flashes in the Absence of Added Substrates

**Figure 4:**
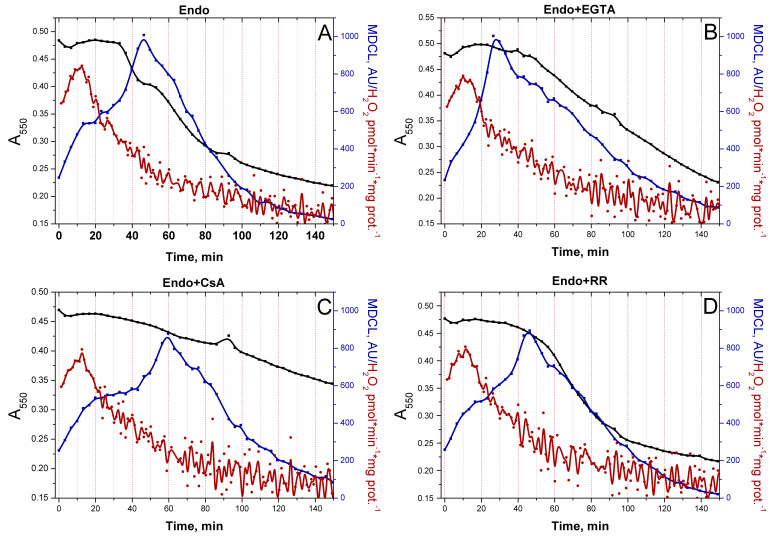
Effect of mPTP inhibitors on the O_2_^−^/H_2_O_2_ release from RLM oxidizing Endo substrates. RLM (0.75 mg protein/mL) were treated exactly as described in Figure 2. Where indicated, the wells also contained 1 mM EGTA (**B**), 1 μM CsA (**C**), 2 μM RR (**D**), or nothing (**A**). In all panels, black, red, and blue lines indicate changes in absorbance, H_2_O_2_ production, and MDCL, respectively. Points on the curves are the means (*n* = 3) of three technical replicates. The figure shows one representative experiment of at least five similar ones.

In order to discriminate the role of Ca^2+^ and of mPTP opening per se in the persistence of O_2_^−^ bursts in RLM that oxidize Endo substrates in the presence of EGTA (Figure 3B), we studied the effect of the other mPTP antagonists (CsA and RR) on the kinetics of swelling and O_2_^−^/H_2_O_2_ release (Figure 4). In all cases, the highest rate of H_2_O_2_ release was noted in the first minutes of incubation followed by the inhibition of H_2_O_2_ production. Since mPTP inhibitors did not prevent the suppression of H_2_O_2_ release, the suppression might be a consequence of the exhaustion of Endo substrates and/or the mild uncoupling (Supplementary Figure S1), which declined the extent of the reduction of ETC redox centers. The decline in H_2_O_2_ generation preceded the activation of O_2_^−^ release. O_2_^−^ bursts could occur before high-amplitude mitochondrial swelling when mPTP opening was blocked by EGTA, CsA, and RR (Figure 4B–D). Therefore, respiratory substrates can limit the O_2_^−^ release from mitochondria independently of mPTP opening. Hence, the presence/absence of a respiratory substrate can be another trigger of the switching of the type of a ROS signal emitted by mitochondria (when the mPTP is closed).

### 3.5. Modulation of the Type of a ROS Signal by Different Respiratory Substrates

**Figure 5:**
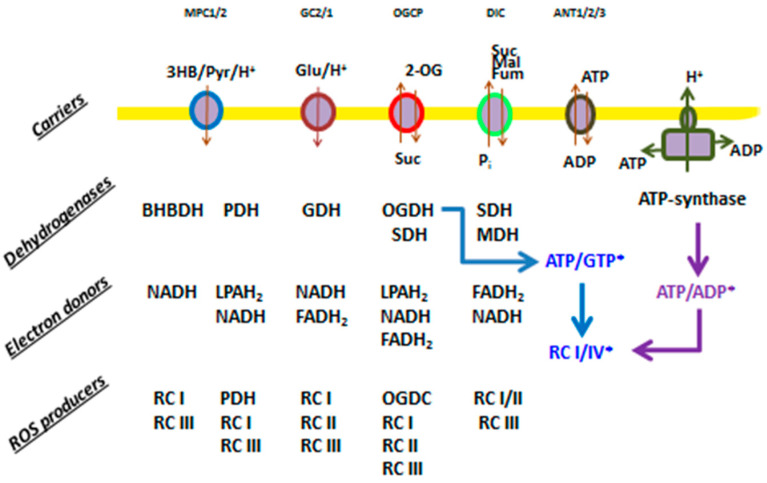
ROS producers in mitochondria supplemented with different respiratory substrates. Carriers: MPC, mitochondrial pyruvate carrier; GC, mitochondrial glutamate carrier; OGCP, mitochondrial 2-oxoglutarate/malate carrier protein; DIC, mitochondrial dicarboxylate carrier; and ANT, adenine nucleotide translocator. Dehydrogenases: BHBDH, 3-hydroxybutyrate dehydrogenase; PDH, pyruvate dehydrogenase; OGDH, oxoglutarate dehydrogenase; SDH, succinate dehydrogenase; MDH, malate dehydrogenase; and GDH, glutamate dehydrogenase. ETC complexes: RCI, RCII, RCIII, and RCIV. Electron donor: LPAH_2_, reduced lipoamide. The asterisk shows indirect modulators of ROS production.

Different respiratory substrates are transported into the matrix by appropriate carriers via the symport with H^+^ or electrically neutral exchange with an anion and are oxidized by specific dehydrogenases (Figure 5). The resulting set of reduced electron donors can donate electrons to several ROS-generating sites, depending on the combination of substrates. The switching of the type of the emitted ROS signal can be associated either with a change in the rate of the production of O_2_^−^/H_2_O_2_ in the same or different mitochondrial redox centers or with an alteration in the efficiency of their release. For H_2_O_2_, it is known that conditions promoting its formation in mitochondria also contribute to the maintenance of the systems of its scavenging in the active state [55]. Therefore, it can be assumed that the rate of the release of H_2_O_2_ is approximately proportional to the rate of its production. The mechanism of O_2_^−^ release from the matrix of intact mitochondria is poorly understood. Acidic pH can dramatically increase the capability of O_2_^−^ for penetration through phospholipid membranes [56,57]. Indirect evidence indicates that, at slightly alkaline pH of the mitochondrial matrix, the release of O_2_^−^ may require the operation of carriers of anionic substrates [58,59,60].

**Figure 6:**
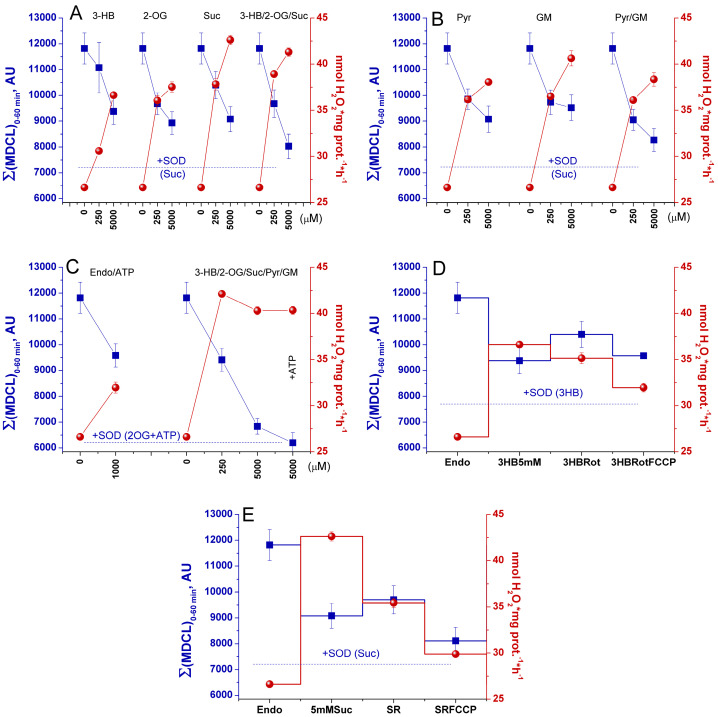
The opposite dose-response effect of respiratory substrates on the release of O_2_^−^ and H_2_O_2_ from mitochondria. RLM (0.4 mg protein/mL) were placed in the standard KCl-BM without added respiratory substrates but supplemented with 1 μM CsA, and the suspension was processed as described in the legend to Figure 3. Where indicated, the wells contained SOD (100 U/mL), respiratory substrates at either 250 μM or 5 mM concentrations: 3-HB, 2-OG, and Suc (**A**); Pyr, GM (**B**); 1 mM ATP, all substrates (**C**); 5 mM 3-HB, 2 μM rotenone (Rot), 500 nM FCCP (**D**); 5 mM Suc, Rot, FCCP (**E**). In all panels, blue and red symbols show the cumulative MDCL (Σ(MDCL)_0–60 min_, AU) and H_2_O_2_ production in the first hour of incubation, respectively. Blue dotted lines show MDCL in the presence of SOD and indicated substrates. The left ordinate axis on all panels starts with the value of MDCL in the presence of SOD and all substrates at a concentration of 5 mM. Experimental points are the means ± S.D. (*n* = 3) of three technical replicates. The figure shows one representative experiment of at least three similar ones.

Therefore, we studied the effect of various respiratory substrates at different concentrations and their combinations on the emission of O_2_^−^/H_2_O_2_ from mitochondria (Figure 6, Supplementary Figure S3). In order to distinguish the own effects of substrates on ROS emission from the effect on mPTP opening, the incubation medium contained the mPTP inhibitor 1 μM CsA, and membrane intactness was monitored as indicated in the legend to Figure 3 and Figure 4. Figure 6A–C and Supplementary Figure S3 show that the addition of any exogenous substrates, especially Suc, at near-physiologic concentrations (250 μM) was sufficient to sharply increase the release of H_2_O_2_ from mitochondria. The most intense production was observed in the presence of combinations of the OXPHOS substrates 3-HB/2-OG/Suc (Figure 6A) and 3-HB/2-OG/Suc/Pyr/GM (Figure 6C). A rise in the substrate concentrations to 5 mM (saturation) caused an increase in H_2_O_2_ generation in the presence of all substrates except the combination of six substrates (Figure 6C). Thus, the mixture of six substrates at near-physiologic concentrations caused the reduction of redox centers sufficient for maximum production of H_2_O_2_. The decrease in H_2_O_2_ release with the mixture of 5 mM substrates is obviously due to the competition between the substrates for coenzymes, the bidirectional transport of substrates and intermediates, and the different activities of transporters and dehydrogenases of different substrates (Figure 5). In all cases, the O_2_^−^ release decreased with increasing substrate concentration regardless of whether the substrate was symported with H^+^ or not. The decrease in MDCL contrasted with the increase in H_2_O_2_ production. A noteworthy exception was the presence of the six-substrate combination, where the maximum boost and the slight decrease in H_2_O_2_ production at 250 μM and 5 mM substrate concentrations, respectively, coincided with a moderate and an almost complete suppression of O_2_^−^ liberation (Figure 6C). These data indicate that the carriers of anionic substrates may contribute to the O_2_^−^ release from the matrix and that the transport of substrates competes with this process.

The mechanism of suppression of the O_2_^−^ efflux by respiratory substrates may involve a switch in the redox centers and an alkalization of the matrix. Therefore, we studied the effect of the CI inhibitor rotenone and the protonophore FCCP on the rate of O_2_^−^ and H_2_O_2_ release in the presence of substrates of CI and CII. The oxidation of Endo substrates in the forward electron transport (FET) mode (CI-CIII-CIV segment of ETC) yielded maximum O_2_^−^ and minimum H_2_O_2_ (Figure 6D,E). An additional reduction in CI (and, subsequently, CIII) with 3-HB accelerated the generation of H_2_O_2_ and diminished the O_2_^−^ release. Rotenone, which induces the complete reduction of the flavin site of CI and the oxidation of the Q-CIII-CIV segment of the ETC accompanied with the disruption of the H^+^ gradient across the IMM, moderately stimulated the O_2_^−^ release and slightly suppressed H_2_O_2_ production, indicating the major contribution of the CI flavin site to the H_2_O_2_ production (Figure 6D). In the case that the addition of Suc activated both FET (CII-CIII-CIV segment) and reversed electron transport (RET) (CII-CI segment), the release of H_2_O_2_ was strongly enhanced, while the O_2_^−^ release was suppressed even more strongly than upon FET (Figure 6E). Rotenone, which stops RET without affecting the FET and the H^+^ gradient, strongly suppressed the H_2_O_2_ efflux and had a minor effect on the O_2_^−^ emission. FCCP, which causes a complete oxidation of ETC downstream the rotenone block and the dissipation of the H^+^ gradient across the IMM, restrained the generation of O_2_^−^/H_2_O_2_ on both substrates, though to a different extent. These results indicate that the switching of the type of ROS signal is associated primarily with a change in the extent of the reduction of ETC redox centers, but not with the H^+^ availability in the matrix. The type of signal emitted by mitochondria corresponds to a pattern of ROS production as if fully reduced redox centers generated H_2_O_2_, partially reduced ones generated O_2_^−^, and oxidized ones generated nothing.

### 3.6. Effect of Respiratory Substrates on the Spontaneous and Xanthine Oxidase-Dependent Generation of O_2_^−^

**Figure 7:**
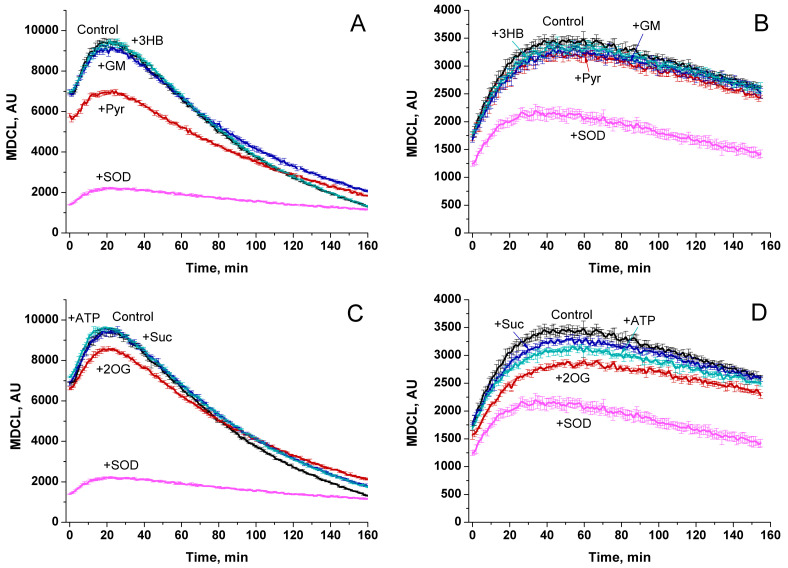

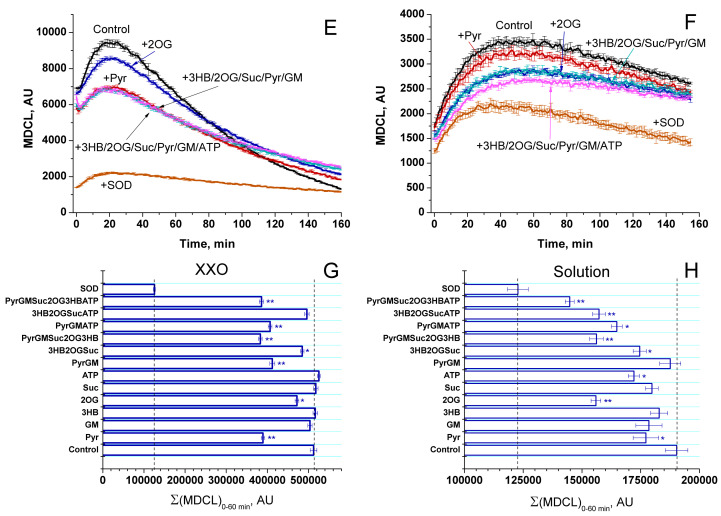
Effect of respiratory substrates on the spontaneous and XO-dependent O_2_^−^ generation. Standard incubation medium was supplemented with 15 μM MCLA (**A**–**H**) and 400 μM xanthine plus XO (0.005 U/mL) (XXO) (**A**,**C**,**E**,**G**), extensively mixed, and immediately added to the wells of a 96-well plate containing, where shown, 1 mM ATP, Pyr, GM, Suc, 3-HB, 2-OG (all at a concentration of 5 mM), and SOD (200 U/mL). (**A**–**F**). Original traces of one representative experiment of three similar ones. Points on the curves are the means ± S.D. (*n* = 3) for three technical replicates. (**G**,**H**) Cumulative MDCL (±cumulative S.D.) within 60 min of incubation for the curves presented in panels (**A**–**F**). Asterisks show the significant difference with the control (*—*p* < 0.05, **—*p* < 0.01).

Another possible mechanism for the suppression of O_2_^−^ release from mitochondria by respiratory substrates is the direct antioxidant effect of the latter. Figure 7 shows the effect of different OXPHOS substrates (all at a concentration of 5 mM except ATP (1 mM)) on the spontaneous and xanthine/xanthine oxidase-dependent O_2_^−^ generation (SOD-sensitive MDCL) in aqueous solution. At a near-physiologic concentration (250 μM), neither individual substrates nor their combinations affected the MDCL. As follows from the data presented, Pyr and to a lesser extent 2-OG decreased the O_2_^−^ level in the xanthine/xanthine oxidase system (Figure 7A,C,E,G). Spontaneously generated O_2_^−^ was scavenged by 2-OG, and to a lesser extent ATP and Pyr (Figure 7B,D,F,H). In contrast, 3-HB, Suc, and GM, as well as fumarate and malate, added separately, had a minimal effect on the O_2_^−^ level. These data indicate that alpha-keto acids can operate as the weak O_2_^−^ scavengers (Supplementary Table S1), while other substrates tested cannot.

### 3.7. Effect of Adenine Nucleotides on the O_2_^−^/H_2_O_2_ Release from RLM

**Figure 8:**
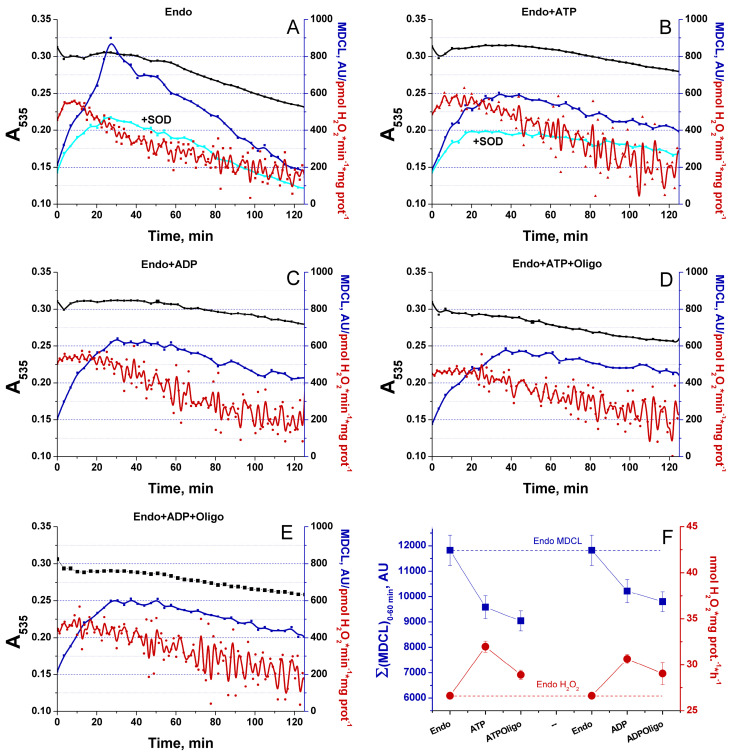
Effect of ANs on the O_2_^−^/H_2_O_2_ release from RLM oxidizing Endo substrates. RLM (0.4 mg protein/mL) were treated exactly as indicated in the legend to Figure 3, except that 1 μM CsA was added to the incubation medium. Were indicated, the wells also contained 1 mM ATP (**B**,**D**), 1 mM ADP (**C**,**E**), 2.5 μM oligomycin (**D**,**E**), and SOD (100 U/mL). In all panels, black, red, and blue lines indicate the changes in absorbance, H_2_O_2_ production, and MDCL, respectively. In panels (**A**,**B**), cyan lines show the MDCL in the presence of SOD. Panel (**F**) shows the data on cumulative MDCL and H_2_O_2_ production per hour. Points on the curves are the means (*n* = 3) of three technical replicates. The figure shows one representative experiment of at least three similar ones.

Then, we explored the effect of added adenine nucleotides (ANs) on the O_2_^−^/H_2_O_2_ release from RLM (Figure 8). ANs are known to strongly inhibit the opening of mPTP [45]. In our preparations, 1 mM ADP and 1 mM ATP increased the calcium retention capacity of RLM oxidizing Endo substrates from 45 to 195 and 240 nmol Ca^2+^ per mg protein, respectively. In order to discriminate the effect of ANs on the O_2_^−^/H_2_O_2_ release from the effect on the mPTP opening, the medium contained 1 μM CsA, and the IMM intactness was monitored as indicated in the legends of Figure 3 and Figure 4. As follows from the figure, ANs considerably suppressed the mitochondrial swelling and O_2_^−^ release but prolonged the H_2_O_2_ emission, probably, due to the support of mitochondrial coupling and the reduced state of mitochondrial redox centers (Figure 8A–C,F, Supplementary Figure S1). The inhibitor of F_O_F_1_-ATP synthase oligomycin slightly enhanced the AN-dependent suppression of O_2_^−^ release. Simultaneously, it stabilized the H_2_O_2_ production at a lower level than in the presence of ANs alone (Figure 8D–F). This may be connected with the allosteric effect of ANs on the ETC complexes. Thus, changes in mitochondrial coupling without mPTP opening and changes in the levels of substrates may contribute to a switch of the ROS-type signal emitted from mitochondria.

## 4. Discussion

In the vast majority of studies devoted to the role of ROS in redox signaling and various physiological and pathological processes, the authors do not discriminate between the effects of O_2_^−^ and H_2_O_2_, considering O_2_^−^ exclusively as a relatively short-lived precursor of stable H_2_O_2_. Even in cases that the production of both species or even O_2_^−^ alone was measured, the authors prefer to use the umbrella term “ROS” [8]. A feature of the experimental approach in this study was the parallel recording of the long-term kinetics of O_2_^−^ and H_2_O_2_ release in a mitochondrial suspension with the simultaneous assessment of the permeability of the IMM to low-molecular-weight compounds (Figure 3, Figure 4, Figure 6 and Figure 8). This allowed exploring the O_2_^−^/H_2_O_2_ release in the context of redox signaling. The O_2_^−^ probe MCLA is well suited for the long-term registration of O_2_^−^ production in a mitochondrial suspension [42,54,61,62,63]. It is very sensitive to O_2_^−^ and can also recognize singlet oxygen [51,53,64]. Since O_2_^−^ flashes are usually accompanied by fluctuations in ΔΨm, the lack of charge in both MCLA radical and its O_2_^−^ adduct [48,53] is a great advantage compared to lucigenin [65] and the oxidized products of hydroethidine (Mito-SOX), ethidium, and 2-hydroxyethidium [66]. MCLA detects O_2_^−^ in suspension between mitochondria (SOD-sensitive MDCL, MDCL_Ext_) (Figure 1, Figure 3, Figure 4, Figure 6 and Figure 8) and in the intermembrane space (SOD-insensitive, OXPHOS substrate-sensitive MDCL, MDCL_IMS_) (Supplementary Figure S4) but not in the matrix of intact mitochondria (Figure 2). The production of H_2_O_2_ using AR/HRP is usually measured in the initial period of the process [40,67,68], but this technique also allows recording the long-term kinetics of H_2_O_2_ production by mitochondria (Figure 1, Figure 3, Figure 4 and Figure 8). Though resorufin accumulation interfered with the recording of both swelling and MDCL, the use of two plate readers in parallel allowed us to solve this problem.

The main finding of this study is that changes in the physiological state of mitochondria lead to changes not only in the rate of ROS release from mitochondria, but also in the type of the ROS signal (or more broadly, the redox signal) emerging from the organelles (Figure 1, Figure 3 and Figure 6, and Supplementary Figure S3). This conclusion, important in the context of redox signaling, raises two questions. First, what is the mechanism for the switching of the type (O_2_^−^ or H_2_O_2_) of the outgoing redox signal? Second, what is the possible physiological role and the pathological effect of this switch? It is obvious that two main mechanisms of the observed switching of the type of ROS signal are possible: a real switching in the redox centers of mitochondria and an apparent switching due to the facilitation of the release of O_2_^−^ from mitochondria (mPTP/pore-dependent or -independent mechanism).

According to modern concepts, the majority of mitochondrial ROS-generating enzymes and complexes produce ROS as a result of one-electron leaks in flavin- and Q-binding sites with the formation of O_2_^−^ (CI, CII, and CIII, dihydroorotate dehydrogenase, glycerol-3-phosphate dehydrogenase, electron-transporting flavoprotein) [69,70,71,72,73], which subsequently dismutate to H_2_O_2_. These views were based on the data of early studies of ROS in mitochondria. The studies conducted using complex III inhibitors, which stabilize semiubiquinone at one of the binding sites, led to the conclusion that the only precursor of all ROS in mitochondria is O_2_^−^ [74,75,76]. In this case, a necessary condition for the maximum generation of O_2_^−^ and, as a consequence, H_2_O_2_, is the maximum reduction in the ETC segment that ends with the semiquinone form of flavin or of ubiquinone [74,76].

If this model is the only correct one, then the only possibility of switching the type of ROS signal exiting the mitochondria would be a disproportional facilitation of the exit of one of the ROS types (O_2_^−^) through mPTP (or another pore) or by another mechanism. However, there is evidence indicating that this model is not universal. In inside-out submitochondrial particles, in which there are no restrictions on the release of ROS, it was shown that 1 mM NADH suppresses the generation of O_2_^−^ in both FET and RET (10 mM Suc) in the presence of rotenone [67]. Moreover, the dependence of the O_2_^−^ production rate on the NADH concentration is bell-shaped, with the maximum rate at a concentration of about 50 μM, while the maximum rate of H_2_O_2_ generation (mainly in CI) occurs at a NAD(H) concentration of ~100–500 μM and the maximum NADH/NAD+ ratio [40,68]. The same pattern of H_2_O_2_ production was observed for isolated dihydrolipoamide dehydrogenase [40]. It is important that, at low NADH concentrations, the only type of ROS generated in submitochondrial particles and isolated CI is O_2_^−^, while at high NADH levels, the main type of ROS becomes H_2_O_2_, which, according to the authors, should be the main type of ROS at physiological substrate concentrations [67]. We previously demonstrated that, in mitochondria with the IMM permeabilized due to mPTP opening or the incorporation of a pore-forming peptide, an O_2_^−^ burst occurs after significant oxidation of the added substrates NADH or NADPH (positive shift in *E*_NAD(P)H_) [42]. These data indicate the possibility of a real switch in the type of ROS generated, at least in mitochondrial complex I.

It should be mentioned that initially, we suggested that NADPH-dependent O_2_^−^ bursts are associated with the activity of adrenodoxin-adrenodoxin reductase complex [42]. However, since O_2_^−^ bursts are not detected in mitochondria lacking functional ETC complexes [10], and CI can oxidize NADPH at a low rate [77], it can be assumed that CI is also responsible for the NADPH-dependent generation of O_2_^−^. At the same time, in intact mitochondria, succinate, which is devoid of antioxidant properties (Figure 7, Supplementary Table S1), potently suppressed O_2_^−^ generation (Figure 1, Figure 3 and Figure 6 and Supplementary Figure S3), and rotenone had a minor effect on this process (Supplementary Figure S4). This suggests the participation of other, non-CI, partially reduced electron carriers of the CII-CIII-CIV segment of ETC in the generation of O_2_^−^.

In this work, we confirm the data of other groups [39,40,68] indicating that, in intact mitochondria, the rate of H_2_O_2_ generation is maximum at the maximum coupling (Figure 1 and Figure 8) and the maximum concentration of respiratory substrates (Figure 1, Figure 3 and Figure 6 and Supplementary Figure S3). This mirrors the situation with the release of O_2_^−^, which maximum rate was observed at low (near-physiological) substrate concentrations, on Endo substrates and upon reduced coupling (Figure 4, Figure 6 and Figure 8, and Supplementary Figure S1) [78,79]. Thus, these data confirm that the switching of the ROS signal type during changes in the functional state of intact mitochondria occurs in redox centers. mPTP induction (or creation of any other pore in the IMM) not only opens the way for the free release of O_2_^−^ (Figure 3), but also causes oxidation of the mitochondrial redox centers, the degree of which depends on the concentration of available substrate (Supplementary Figure S1) [42].

The mechanism of switching the type of ROS signal may be different in redox centers of different complexes. As for complex I, its electron transfer pathway consists of terminal two-electron carriers FMN and ubiquinone and intermediate one-electron-transferring FeS clusters (N3, N1b, N4, N5, N6a, N6b, and N2). The chain is organized in such a way that (a) electron transport is slow, (b) electrons can move in both directions, and (c) the chain is reduced in coupled mitochondria [77]. In addition to the main chain of FeS clusters, there are two additional ones, N7 and the highly conserved N1a, which is adjacent to FMN and is thought to play an important role in preventing ROS generation [80]. Since FeS clusters can be inactivated by O_2_^−^ [81,82,83], to prevent self-inactivation of the complex, mechanisms for the elimination of O_2_^−^ or its additional reduction to H_2_O_2_ could have been evolutionarily developed. Although the presence of O_2_^−^ in the membrane is extremely thermodynamically unfavorable [84], the membrane can be a buffer for the reactive hydroperoxyl radical [56,57,85,86]. Therefore, additional reduction of solvated O_2_^−^ to H_2_O_2_ is not only thermodynamically beneficial (Gibbs energy of formation of O_2_^−^ and H_2_O_2_ in aqueous solutions (Δ*G*^0^) are +7 and −134 kJ/mol; standard electrode potentials (*E*^0^) are −0.18 and + 0.36 V, respectively [87,88]), but also rational from the point of view of preserving the activity of the complex. It can be assumed that in coupled mitochondria at a high NADH/NAD^+^ ratio, the probability of electron return to partially reduced FMN (or ubiquinone) and two-electron reduction of oxygen to H_2_O_2_ increases. In contrast, partial oxidation of electron carriers (Figure 6D,E) may decrease the probability of O_2_^−^ reduction by second electron and thus promote the production of O_2_^−^ by both intact and permeabilized mitochondria.

The question arises whether there is any contribution of the facilitated O_2_^−^ exit via mPTP or alternative mechanism to the switching of the type of emitted signal? In the presence of Endo substrates, the decrease in the level of H_2_O_2_ generation is accompanied by nearly the same activation of O_2_^−^ generation, regardless of whether the decline in H_2_O_2_ generation was accompanied by mPTP opening or not (Figure 4). Thus, the contribution of mPTP opening per se to the activation of O_2_^−^ output appears to be small.

Theoretically, there may be at least two mPTP-independent mechanisms of the facilitated O_2_^−^ release from the mitochondrial matrix. The first should involve a protonation of O_2_^−^ (anion) to neutral hydroperoxyl radical (pKa = 4.8) [87,88], which penetrates through phospholipid membranes even more effectively than H_2_O_2_ [56,57]. The efficient protonation requires the decline in the matrix pH from the physiological values of 7.8–8.0 to 7.1–7.3 (common to the pH of cytosol) or even lower. The second mechanism, implied by a body of indirect evidence [58,59,60], should include the extrusion of O_2_^−^ by the transporters of anionic substrates.

Since neither protonophore FCCP nor substrates transported to the matrix via the symport with protons (in comparison with other substrates) increased the release of O_2_^−^ produced by the whole ETC or its segments either before or after the rotenone block (Figure 5 and Figure 6), O_2_^−^ protonation, presumably, has a minor effect on the activation of the O_2_^−^ efflux in our experimental model. At variance, the dose-dependent effect of the combination of substrates on the O_2_^−^/H_2_O_2_ release (Figure 6C) supports the possibility of O_2_^−^ extrusion by the carriers of anionic substrates, though this question requires a more detailed study.

It should be stressed that the dose-dependent decline in the level of the O_2_^−^ by all respiratory substrates tested but alpha-keto acids (Pyr and 2-OG) (Figure 3 and Figure 6) is not related to their O_2_^−^-scavenging ability (Figure 7, Supplementary Table S1). Moreover, though alpha-keto acids can operate as the weak O_2_^−^ scavengers, one still can observe O_2_^−^ bursts in their presence under certain conditions (Figure 3), which indicates that their scavenging effect cannot cancel strong O_2_^−^ generation in redox centers.

The physiological or pathophysiological significance of the switching of the type of ROS signal emitted by mitochondria becomes more understandable when considering the modern data on the organization of the redox signaling system in a cell [3,89]. This system includes the subsystems of ROS generation (more than 40 enzymes and complexes), transformation of the ROS/redox signal (SOD, GSH-peroxidase, nitric oxide synthase, etc.), signal attenuation/amplification (cytochrome c, catalase, GSSG reductase, enzymes supporting ROS-induced ROS release), ROS/redox-dependent adapters (thioredoxins, glutaredoxins, peroxiredoxins, etc.), and ROS/adapter-dependent effectors (protein kinases, phosphatases, ion channels, transcription factors) [3,89,90,91,92,93,94]. Among ROS generated in mitochondria, the hydroxyl radical is the most reactive and short-lived (10^−9^ s) [95], which strongly limits the range of its activity. By contrast, H_2_O_2_, stable and capable of penetrating through membranes, is considered to be the main signaling ROS, which links the subsystems of redox signaling in the cell [3,89]. Not much is known about the own, H_2_O_2_-independent, role of O_2_^−^ in the redox signaling. On the one hand, being an anionic reductant scarcely penetrating through membranes [85], it should act locally: inside or in close proximity to mitochondria. In particular, O_2_^−^ can reversibly inhibit FeS-containing proteins like aconitase, CI, and CII [81,82,83], and, to some extent, adenine nucleotide translocase [96]. This, in turn, can modify ionic homeostasis and cristae morphology [97]. On the other hand, protonated O_2_^−^ (hydroperoxyl radical) can easily escape from mitochondria or propagate within membranes [56,57,85,86]. This should increase the range of its effectiveness as a redox signal transmitter, although it is not clear to what extent. It is also unclear whether O_2_^−^- and hydroperoxyl radical-specific adapters and effectors exist beyond mitochondria. At the same time, a body of indirect evidence indicates that an O_2_^−^/hydroperoxyl radical-dependent subsystem of redox signaling may exist. Indeed, O_2_^−^, directly or through a local intermediary, can affect many intracellular processes of both physiologic and pathologic nature [9,14,19,23,24,25,26,27,28,29]. It is important that, in some cases, O_2_^−^-mediated redox signaling was clearly distinguished from the H_2_O_2_-mediated one on the level of a whole cell [98,99]. The results of our study indicate that the level of substrates, the degree of mitochondrial coupling, and mPTP induction can switch the type of the ROS signal emitted by mitochondria, which makes it possible to specifically activate O_2_^−^- or H_2_O_2_-dependent signaling cascades.

## Data Availability

The data presented in this study are available on request from the corresponding author.

## Supplementary Materials

The following supporting information can be downloaded at: https://www.mdpi.com/article/10.3390/antiox13111317/s1, Figure S1: Dynamics of membrane potential and redox state of pyridine nucleotides (PN) in mitochondria during prolonged incubation in the presence of mPTP modulator; Figure S2: Effect of respiratory substrates and mPTP inhibitors on the release of O_2_^−^ (A,C,E) and H_2_O_2_ (B,D,F) from RLM; Figure S3: Dose-response effect of dicarboxylates on the release of O_2_^−^ and H_2_O_2_ from mitochondria; Figure S4: Effect of Suc, Rot, and CsA on the kinetics of O_2_^−^ release from intact RLM; Table S1: Suppression of spontaneous and xanthine oxidase-dependent O_2_^−^ generation by respiratory substrates that are alpha-keto acids.
