# Supplementary material for: The Switching of the Type of a ROS Signal from Mitochondria: The Role of Respiratory Substrates and Permeability Transition
Source: Antioxidants (Basel). 2024 Oct 29;13(11):1317. doi: 10.3390/antiox13111317 (PMC11591497; doi:10.3390/antiox13111317)
Supplement: Supplementary file 1 [file antioxidants-13-01317-s001.zip › antioxidants-3256290-supplementary.pdf]

Supplementary Materials

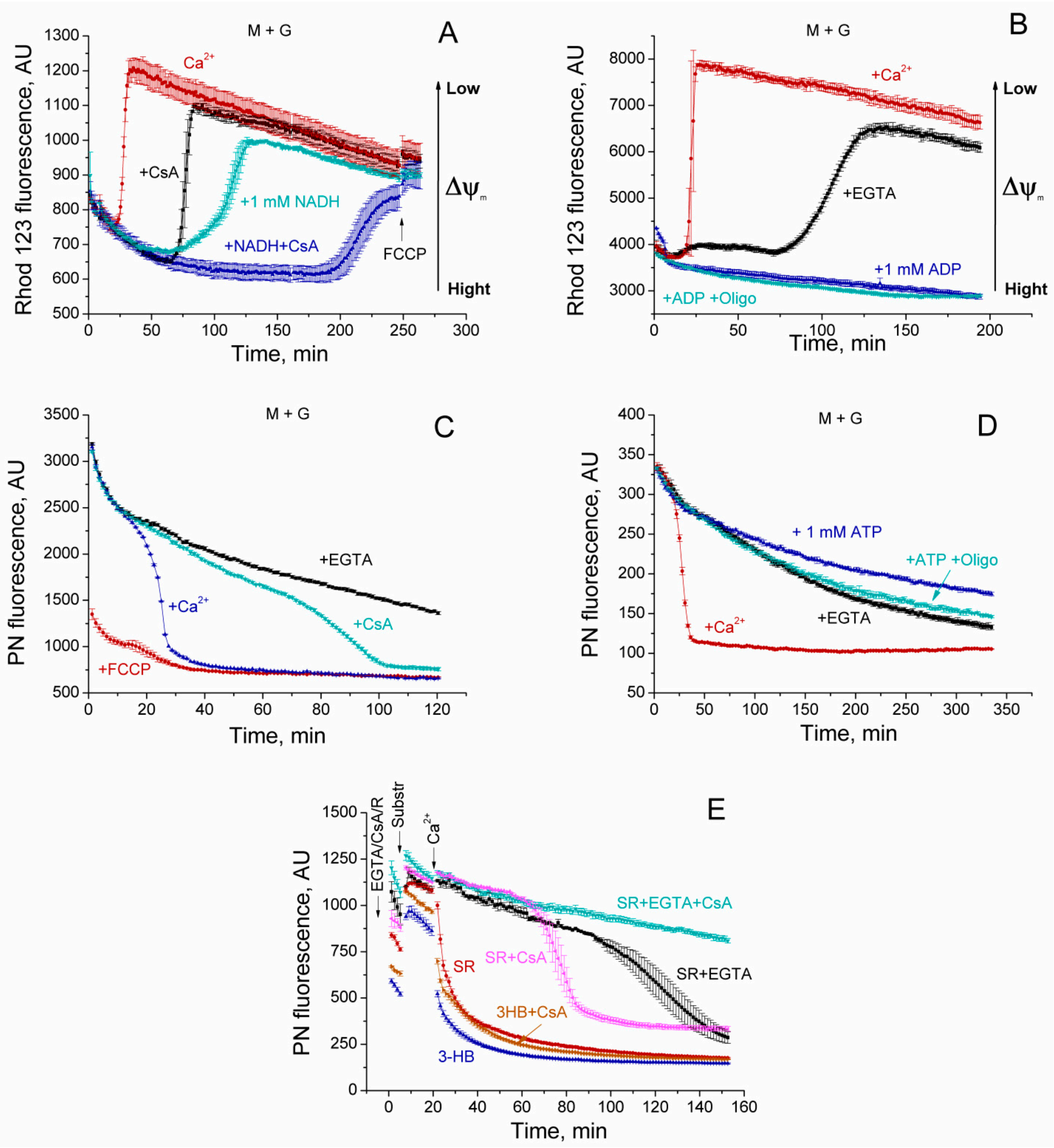

Supplementary Figure S1. Dynamics of membrane potential and redox state of pyridine nucleotides (PN) in mitochondria during prolonged incubation in the presence of mPTP modulator. A and B. Effect of mPTP modulators on the ΔΨm dynamics. The incubation medium contained 0.5 mg/ml of mitochondrial protein, 5 mM malate plus 5 mM glutamate (M+G), 10 μM Ca^{2+}, 330 nM rhodamine 123 (Rhod 123), and, where shown, 1 mM ADP, 1 mM NADH, 1 mM EGTA (EGTA), 5 μg/ml oligomycin (Oligo), and 1 μM cyclosporine A (CsA). C–E. Dynamics of PN fluorescence in a mitochondrial suspension in the presence of different respiratory substrates and mPTP modulators. Where indicated, medium contained 5 mM malate plus 5 mM glutamate (M+G), 1mM EGTA, 1 mM ATP, 5 μg/ml Oligo, 2 μg/ml rotenone (R), 1 μM CsA, and 250 nM FCCP. E. Arrows show the addition of EGTA, CsA, R, substrates (Substr) (5 mM succinate (S) to R-containing samples (SR) and 5mM 3-hydroxybutyrate (3-HB)), and 25 μM Ca^{2+}. The concentration of free Ca^{2+} was 10 (C and D) and ~ 3 μM (E). In panels A and B and C–E, signal acquisition was performed at different gain. Points on the curves are the means of three technical replicates (n = 3). The figure shows representative experiments of at least three similar.

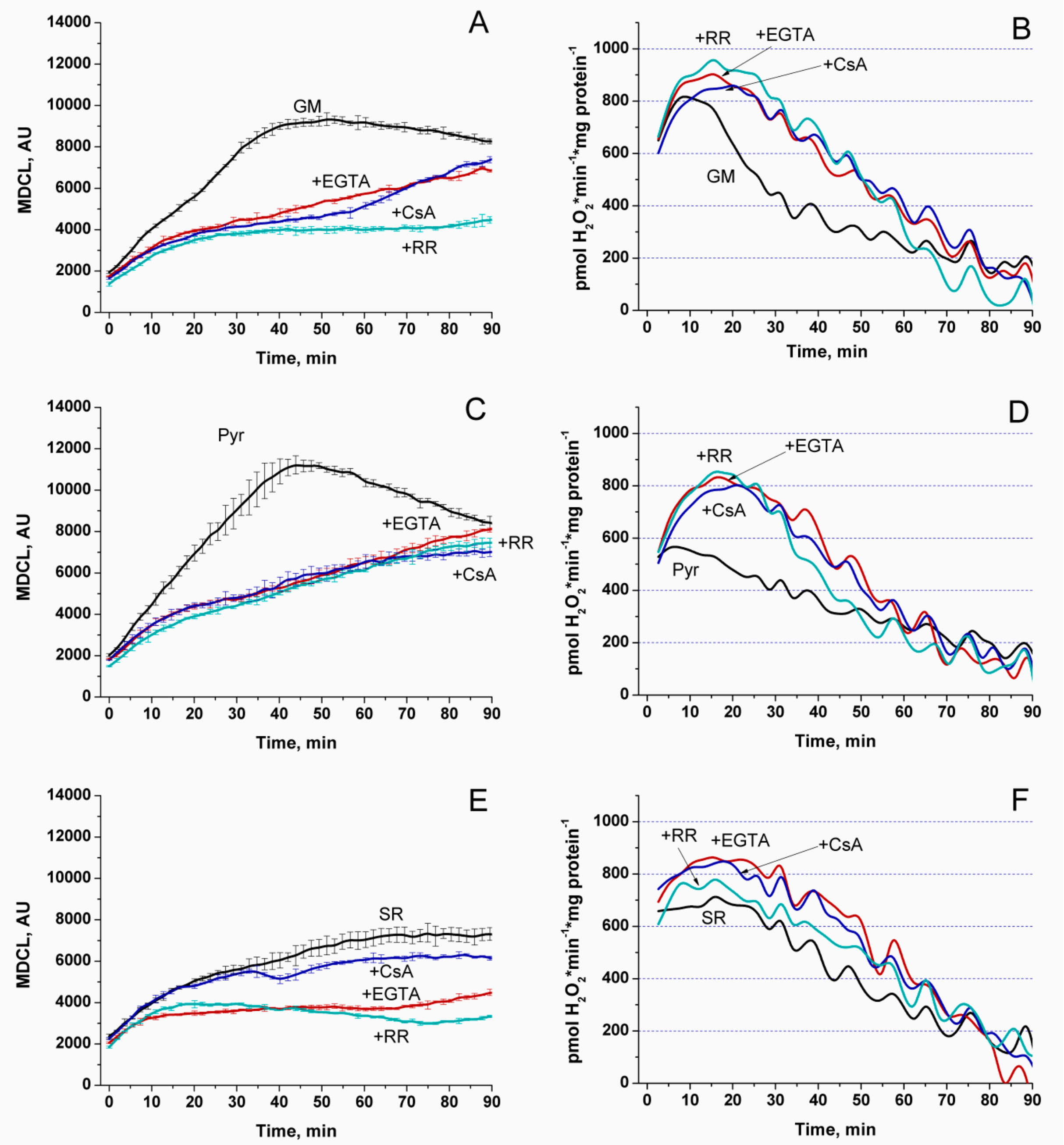

Supplementary Figure S2. Effect of respiratory substrates and mPTP inhibitors on the release of $O_2^{\cdot-}$ (A, C, and E) and H_2O_2 (B, D, and F) from RLM. RLM (0.75 mg protein/ml) were placed in the standard KCl-BM without added respiratory substrates and EGTA, and the suspension was processed as described in the legend to Fig. 1. The wells contained 5 mM GM (A and B), 5 mM Pyr (C and D), 5 mM Suc plus rotenone (2 μg/ml) (SR) (E and F), and, where indicated, 1 mM EGTA, 2 μM RR, and 1 μM CsA. The intensity of MDCL expressed in AU is shown. Points on the curves are the means ± standard deviation (n = 3) of three technical replicates. The rate of H_2O_2 production is expressed in pmol·min^{-1}·mg protein^{-1}. Points on the curves are the means of three technical replicates (n = 3). The figure shows one representative experiment of at least five similar.

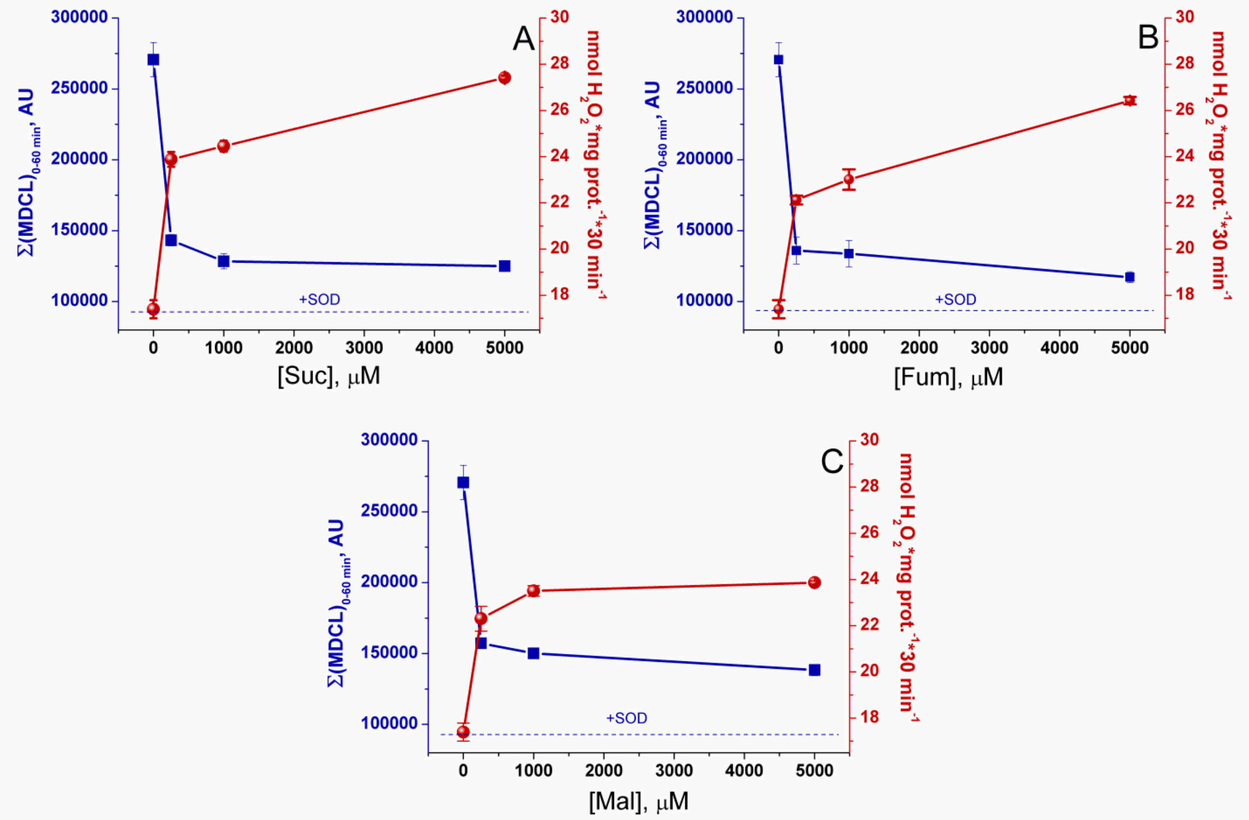

Supplementary Figure S3. Dose-response effect of dicarboxylates on the release of $O_2^{\cdot-}$ and H_2O_2 from mitochondria. RLM (**0.75 mg protein/ml**) were placed in the standard KCl-BM without respiratory substrates but supplemented with 1 μM CsA, and the suspension was processed as described in Fig. 3. Were indicated, the wells contained Suc (**A**), fumarate (Fum) (**B**), malate (Mal) (**C**) at indicated concentrations, and SOD (100 U/ml). In all panels, blue and red symbols show the cumulative MDCL and H_2O_2 production in the first hour of incubation, respectively. Blue doted lines show the MDCL level in the presence of SOD and indicated substrates. Experimental points are the means ± S.D. (n = 3) of three technical replicates. The figure shows one representative experiment of at least three similar.

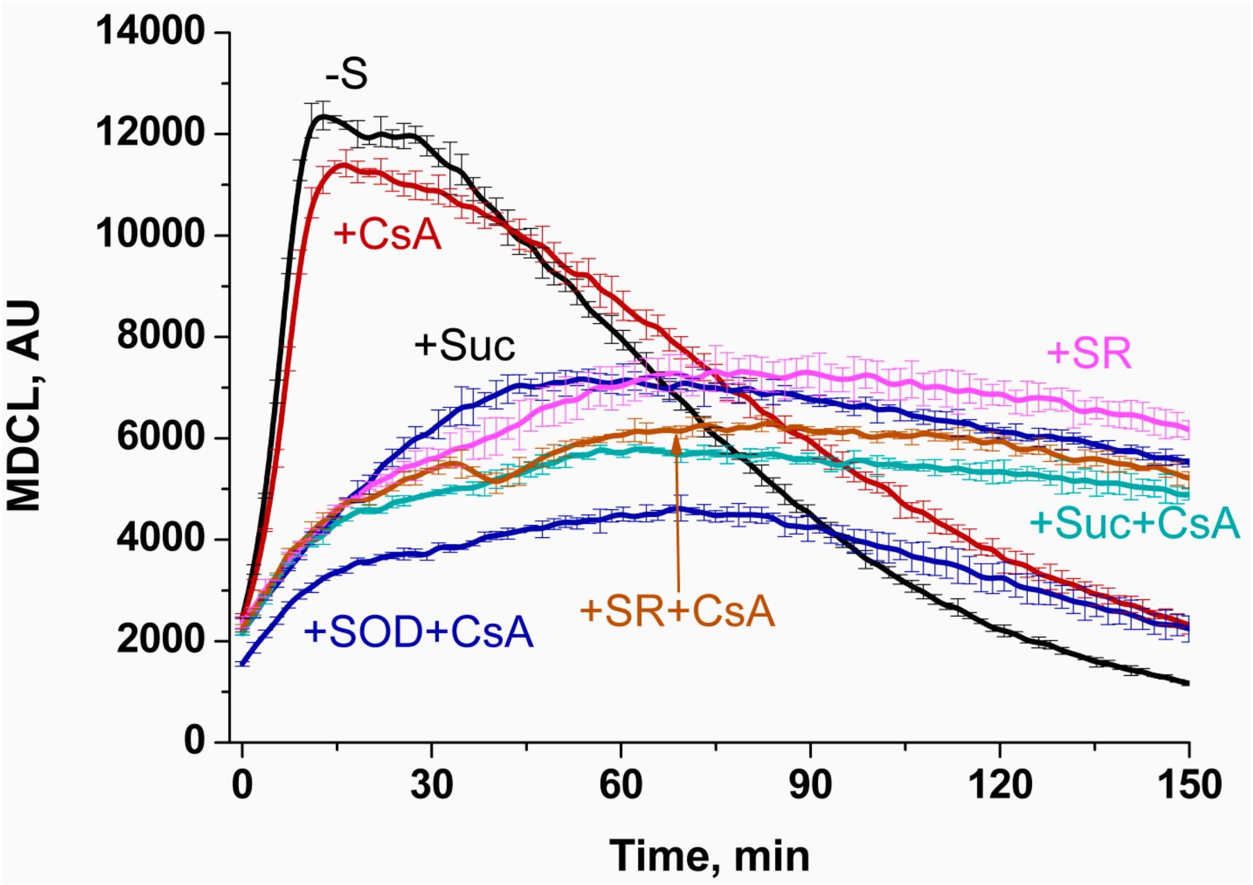

Supplementary Figure S4. Effect of Suc, Rot, and CsA on the kinetics of $O_2^{\cdot-}$ release from intact RLM. Experimental conditions were as in Fig. 6. Concentrations of Suc, Rot, CsA, and SOD were 5 mM, 2 μg/ml, 1 μM, and 100 U/ml, respectively. Experimental points are the means ± S.D. (n = 3) of three technical replicates. The figure shows one representative experiment of at least three similar.

Supplementary Table S1. Suppression of spontaneous and xanthine oxidase-dependent $O_2^{\cdot -}$ generation by respiratory substrates that are alpha-keto acids.

Substrate	Structural formula	XnXnOx	Solution
3-hydroxy butyrate (3-HB)	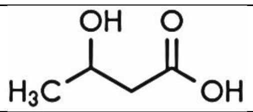	-	-
Pyruvate (Pyr)	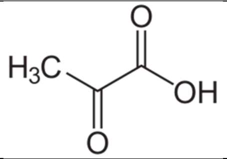	+	+
Succinate (Suc)	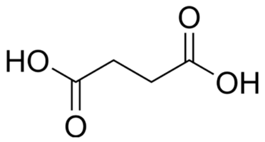	-	-
Fumarate (Fum)	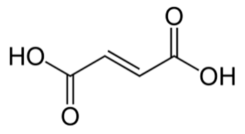	-	-
Malate (Mal)	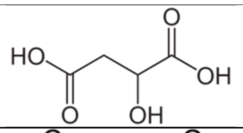	-	-
2 oxoglutarate (2-OG)	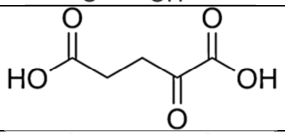	+	+
Glutamate (Glu)	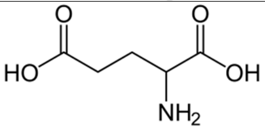	-	-

Note: Original representative traces demonstrating the MDCL suppression are shown in Fig. 7.
